# Phthalates in Animal Husbandry—A Narrative Review

**DOI:** 10.3390/ijms27198746

**Published:** 2026-09-30

**Authors:** Slawomir Gonkowski

**Affiliations:** Department of Clinical Physiology, Faculty of Veterinary Medicine, University of Warmia and Mazury in Olsztyn, Oczapowskiego 13, 10-957 Olsztyn, Poland; slawomir.gonkowski@uwm.edu.pl

**Keywords:** phthalates, farm animals, exposure, environmental pollution, endocrine disruptors

## Abstract

Phthalates (PAEs) are a group of organic compounds widely used across various industries. Their extensive application has led to significant environmental pollution; consequently, they are now ubiquitously found in surface water, groundwater, soil, the atmosphere, and plants. Classified as endocrine-disrupting chemicals (EDCs), PAEs can enter human and animal organisms via the digestive tract, respiratory system, and skin, exerting multidirectional harmful effects. An important yet relatively poorly understood issue is the exposure of livestock to these substances. These compounds—primarily di(2-ethylhexyl) phthalate (DEHP)—can adversely affect various organs of farm animals and transfer into products of animal origin. Although previous studies have demonstrated the presence of PAEs in different tissues of farm animals and raw animal products, current knowledge on this matter remains highly limited. Therefore, further comprehensive research is urgently required to precisely clarify all aspects of livestock exposure to PAEs and to assess the potential consumer hazards associated with their presence in raw, animal-derived products. However, there is no review evaluating the overall impact of PAEs on livestock. This article aims to fill this gap in the literature by providing an overview of current knowledge regarding the sources and levels of exposure across various livestock species, the impact of PAEs on animal health, and the concentration of these compounds in raw products of animal origin. Ultimately, this review may contribute to the advancement of knowledge in this field by facilitating the strategic planning of future research on this critical topic.

## 1. Introduction

**Figure 1:**
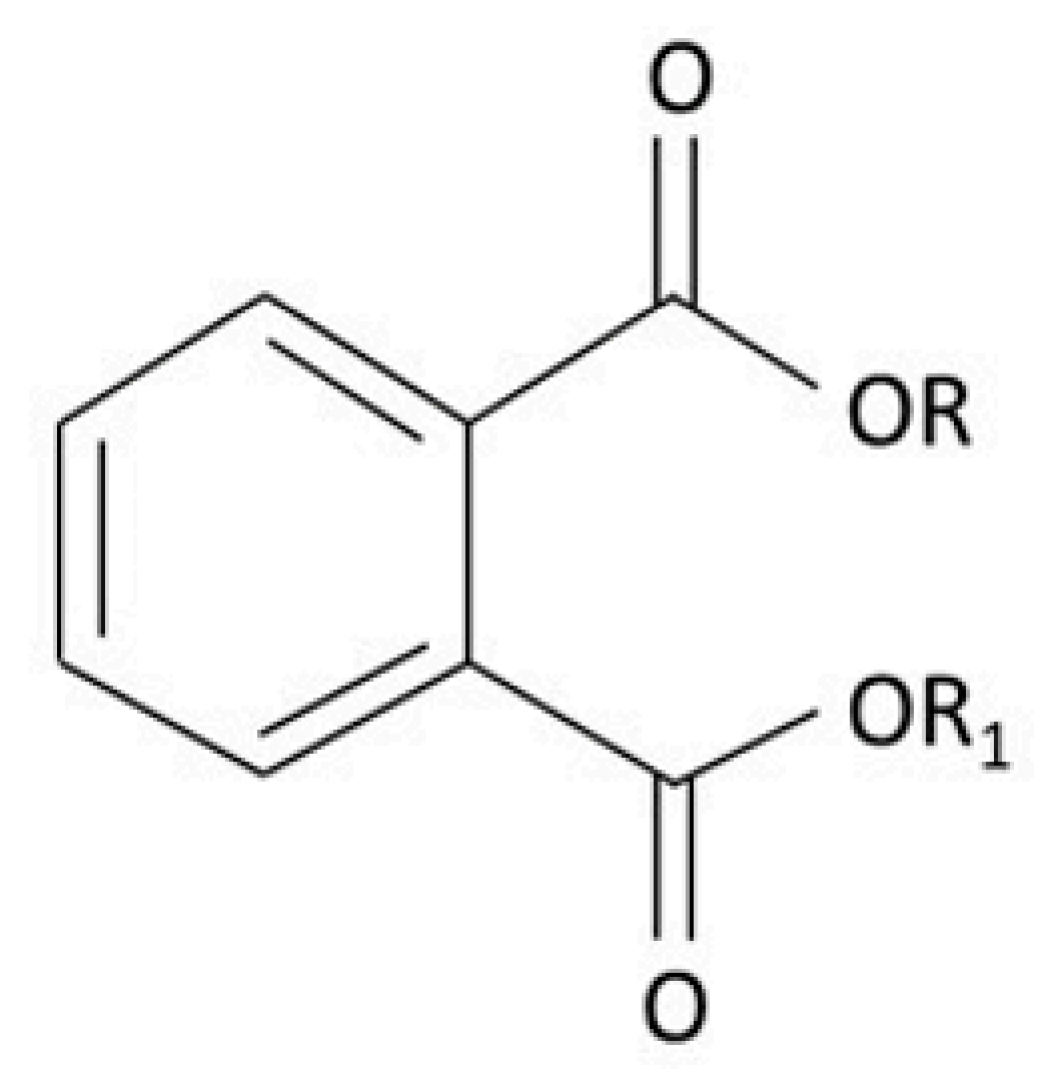
The structure of a phthalate molecule.

Phthalates (also known as phthalic acid esters, PAEs) [Figure 1] are synthetic organic substances, chemically defined as esters of 1,2-benzenedicarboxylic acid, which have been used in various industrial sectors since the 1920s to provide high plasticity, elasticity, and durability [1].

Based on their chemical structure, PAEs are categorized into two types: (1) low-molecular-weight PAEs, which contain short carbon chains (up to 6 carbon atoms) and are often used in personal care products, PVC products, medical devices, and cosmetics; and (2) high-molecular-weight PAEs, which contain longer carbon chains and are commonly used as components of vinyl plastics found in, among other things, household goods, toys, and electronic components [1,2]. It is estimated that the global annual production of PAEs amounts to approximately 8 million tons, and those most widely used in industry are di(2-ethylhexyl) phthalate (DEHP), dibutyl phthalate (DBP), diethyl phthalate (DEP), and diisononyl phthalate (DiNP) [1,3]. The chemical characterization of the PAEs commonly used in industry is presented in the Supplementary Materials (Table S1).

The widespread use of PAEs and the fact that they easily leach from plastics have led to extensive environmental pollution. The presence of PAEs has been detected globally in surface waters, soil, the atmosphere, and plants [4,5,6]. PAEs also infiltrate human and animal bodies through the gastrointestinal tract, the respiratory system, the skin, and—during fetal development—the placenta [7]. Moreover, PAEs can migrate directly into the bloodstream from medical devices such as tubing, blood bags, intravenous cannulas, and catheters [8]. The toxic properties of PAEs are well documented. They are classified as endocrine-disrupting chemicals (EDCs), which affect the functions of the body’s hormone system, thereby leading to the impairment of many internal organs and systems [1]. It has been proven that PAEs exhibit cytotoxic, genotoxic, and carcinogenic properties, influencing, among others, the reproductive, nervous, immune, and cardiovascular systems [1,9,10,11].

Due to these potent, multidirectional toxic effects, the monitoring of living organisms for PAE exposure is of paramount importance in modern toxicology. In human and animal organisms, PAEs are rapidly metabolized through hydrolysis, oxidation, and glucuronidation [12] (Supplementary Materials—Figure S1). Therefore, biomonitoring studies in humans and animals often determine the levels of phthalate metabolites, most commonly the primary ones formed during hydrolysis, which is the first step of phthalate metabolism [13,14]. The PAE metabolites commonly used in biomonitoring studies are presented in the Supplementary Materials (Table S1).

Human exposure to PAEs and its influencing factors are relatively well understood. In contrast, the situation is entirely different for farm animals, where knowledge regarding PAE exposure and its impact on health remains limited. Nevertheless, the exposure of farm animals to PAEs is significant from both veterinary and breeding perspectives. PAEs may negatively affect animal health and productivity, as well as disrupt reproductive functions [15,16]. Furthermore, the exposure of farm animals to PAEs may lead to their accumulation in products of animal origin, posing a potential risk to consumers [17,18,19]. This work provides the first summary of current knowledge on the exposure of farm animals to PAEs, the impact of these compounds on animal health, and their presence in raw animal-derived products. Consolidating this knowledge into a single review appears particularly important in the context of the One Health approach, which posits that human health is inextricably linked to animal health and shared environmental factors [20].

## 2. Literature Review Methodology

The articles included in this review were obtained through a systematic search of the PubMed, Scopus, and Google Scholar databases up to April 2026. Since this manuscript is presented as a narrative review, it was not intended by the author to be a systematic review. Consequently, a formal PRISMA-based (Preferred Reporting Items for Systematic Reviews and Meta-Analyses) study selection was not performed. The literature gathering and screening were conducted independently by a single reviewer (the author). To ensure transparency and a structured approach, duplicate records were removed prior to screening, and the reference lists of the retrieved relevant articles were manually searched for additional eligible studies. The primary literature search relied on the following representative logical combination: (“phthalate” OR “phthalates” OR “phthalate metabolites” OR “DEHP” OR “DBP” OR “DEP” [or other specific phthalate names and abbreviations]) AND (“farm animals” OR “livestock” OR “poultry” OR “chicken” OR “cow” OR “horse” OR “sheep” OR “farm” OR “meat” OR “milk” OR “eggs”). The articles were initially screened based on their title and abstract, and subsequently verified through full-text reading. To meet the inclusion criteria, an article had to describe the exposure of livestock to phthalates, the levels of phthalates and/or their metabolites in livestock organisms (i.e., cows, sheep, goats, pigs, horses, and poultry) or raw animal products (milk, meat, eggs), or the toxicological effects on organs and/or cells isolated from these species. Only English-language articles from peer-reviewed scientific journals were considered. All studies describing the levels and effects of phthalates on the specified animals were included, regardless of the sample size, the concentration/dose of phthalates and their metabolites, the duration of exposure, or whether the research was conducted in vivo or in vitro. Conversely, articles focusing on species other than cows, sheep, goats, horses, pigs, or poultry, as well as their respective products, were excluded.

## 3. Sources of Farm Animal Exposure to Phthalates

**Figure 2:**
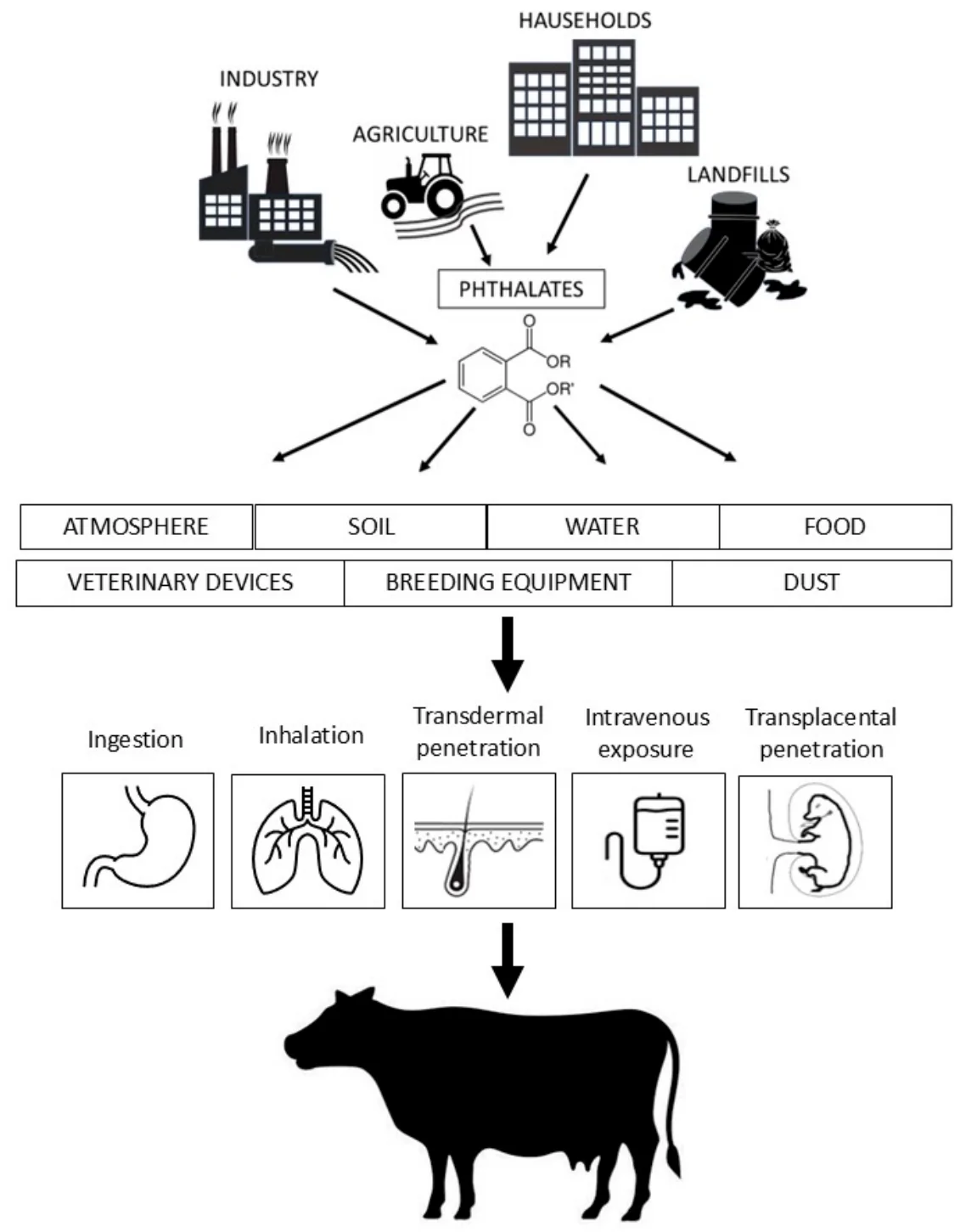
Major sources of phthalate contamination and pathways of their exposure in livestock.

Although the sources of farm animal exposure to PAEs may be highly diverse, they remain poorly understood to date. PAEs enter the environment during plastic production via industrial wastewater, fumes, and dust, as well as through the use of everyday items and household sewage [21,22]. Significant environmental sources also include landfill leachate and agricultural practices—particularly the use of fertilizers and plant protection products, and irrigation with recycled water [23,24,25]. Consequently, PAEs contaminate the atmosphere, soil, feed, surface water, and groundwater, posing a threat to all farm animal species (Figure 2). The primary routes of exposure in humans and animals, which likely also apply to livestock, include ingestion, inhalation, dermal absorption, and direct intravenous entry from medical devices (Figure 2). Moreover, in mammals, transplacental transfer leads to exposure during the prenatal period [26], whereas in birds, PAEs can accumulate in eggs during their formation within the female reproductive tract [18].

The primary route of PAE exposure is dietary intake (food and water), which can account for over 90% of total PAE intake in humans [27]. Naturally, these results should not be directly extrapolated to livestock, as individual species differ significantly in terms of diet composition, soil ingestion, housing conditions, dust exposure, respiratory activity, the potential for dermal contact, and gastrointestinal physiology. However, despite the lack of comprehensive studies on livestock exposure pathways to environmental PAEs, it can be assumed that the dietary route remains highly significant in these animals, as seen in other species, although the extent of exposure may depend on feed composition and digestive tract anatomy (Figure 2). Nevertheless, current knowledge regarding the presence of PAEs in livestock feed remains highly limited. Earlier scarce research was confined to the analysis of PAE concentrations in bovine and porcine feed. Studies, conducted in Belgium, confirmed the contamination of cow feed with DEHP and diisobutyl phthalate (DiBP) across various types of feed, including silage, pasture, and concentrate. The highest levels of these substances (32.92 µg/kg fresh weight and 14.93 µg/kg fresh weight, respectively) were recorded in silage [17]. In contrast, a study on cattle feed conducted in China did not confirm the presence of PAEs [28].

On the other hand, a wide range of PAEs, including DBP, DEHP, benzyl butyl phthalate (BBP), dioctyl phthalate (DOP), DiBP, and dimethyl phthalate (DMP), have been detected in commercial pig feed used in China [29]. DBP and DEHP were found in 100% of the samples, BBP in 91.1%, DOP in 88.9%, DiBP in 13.3%, and DMP in 2.22%. The highest concentrations were observed for DEHP, ranging from 8.26 to as high as 2464 μg/kg (median: 110 μg/kg) [29].

The discrepancies in PAE content in cattle feed between Belgium and China are intriguing [17,28]. It should be noted, however, that both studies were conducted on a very limited number of samples, which could have significantly influenced the results. Therefore, these observations need to be validated using a larger sample size from various farms. Additionally, the variations may stem from differences in methodology, equipment, or detection limits. Alternatively, they may reflect actual differences in feed contamination, arising either from the level of environmental pollution in the studied areas or from different technological processes involved in feed production.

There are two main categories of PAE sources in animal feed. The first relates to the migration of PAEs from the atmosphere, soil, and water into plants during their growth. This is supported by the detection of PAEs in cultivated land, raw cereal grains, and minimally processed feed [17,23]. High levels of these substances in plants may result from the use of fertilizers and plant protection products, as well as the irrigation of fields with recycled water [23,25]. The second category involves contamination during the production process and leaching from packaging materials. The latter phenomenon is relatively well documented [30,31,32]. Additional sources of secondary contamination include troughs and containers used for transport and storage on the farm. Storing feed in non-original containers, such as buckets previously used for paints or varnishes, can be particularly problematic [33]. The relatively low PAE levels found in cereal grains compared to the significantly higher concentrations in packaged and highly processed feed strongly suggest that contamination during production and packaging is the predominant factor [17,34].

Furthermore, oral exposure may occur through drinking water. Previous studies have demonstrated the widespread presence of PAEs not only in surface waters, but also in groundwater and tap water, which are primarily used for watering livestock [4,35]. These levels vary depending on the study location and the type of water. For instance, the mean concentrations of DEHP in surface water and groundwater in a highly industrialized and urbanized area in India can reach up to 2706.135 µg/L and 3203.33 µg/L, respectively [36]. Much lower levels of this substance have been observed in tap water; for example, in Vietnam, the mean level of DEHP in tap water was 5.34 µg/L [37]. Consequently, it can be assumed that drinking water is a significant exposure route. However, surprisingly, the only study investigating PAEs in groundwater on dairy farms did not confirm their presence, which may be due to the very limited number of samples analyzed and/or the type and sensitivity of the method used [17].

Alongside feed and water, soil represents a third, less obvious source of oral PAE exposure for farm animals. Previous studies have shown that annual soil ingestion may reach up to 20 kg in sheep and 180 kg in cows [38,39]. Given that soil is often contaminated with PAEs, this route cannot be overlooked. Observations regarding PAEs in soil concern not only agricultural land but also the immediate environment of dairy farms. Specifically, Fierens et al. [17] detected DMP, DBP, BBP, DEHP, dicyclohexyl phthalate (DCHP), and di(n-octyl) phthalate (DNOP) in soil samples collected from a cattle farm in Belgium. The PAE concentration levels in the farm soil were relatively low: DEHP reached up to 12 µg/kg fresh weight, DBP reached up to 2.67 µg/kg fresh weight, and other PAEs did not exceed 1 µg/kg [17]. However, it should be noted that these studies were conducted on a very limited number of samples (single samples from two farms); thus, higher PAE contamination levels in pasture and farm soils cannot be ruled out.

Indoor dust within farm buildings may also be a significant factor in livestock exposure to PAEs (Figure 2). While direct research on PAE concentrations specifically in farm dust has not been performed, the presence of these compounds in indoor dust is well-established [40]. It is widely recognized that farm animals are highly exposed to dust-bound substances, which enter the body through both the respiratory and digestive tracts [41,42]. Notably, farm dust possesses a unique composition, comprising organic matter from bedding, feed, and animal-derived components such as dander, hair fragments, and particles of excrement [42]. Due to their low vapor pressure, PAEs tend to transition from the gas phase and adsorb onto particulate matter (PM10 and PM2.5) in dust rather than remaining airborne [43,44]. Furthermore, PAEs are lipophilic compounds and have a particular affinity for lipid-rich organic particles [43,45]. Consequently, such dust may serve as a critical vector for PAE exposure in livestock via both the gastrointestinal and respiratory tracts, particularly in animals spending their entire lives in livestock buildings, such as pigs or poultry. This is confirmed by studies that have shown that oral exposure to PAEs via dust significantly increases metabolite levels in the urine of piglets, suggesting that indoor dust is a relevant exposure source in this animal species [46].

Other routes, such as inhalation and transdermal penetration, though well-established in general literature [7], have not yet been specifically investigated in farm animals. Nevertheless, given the ubiquity of PAEs in the atmosphere and their high affinity for organic dust particles [6,43], these pathways likely represent significant exposure routes for livestock. A similar gap exists regarding exposure linked to veterinary treatments. While PAEs are known components of medical devices—such as tubing, intravenous cannulas, and catheters—as well as certain pharmaceuticals [8,47], the specific impact of veterinary procedures on PAE levels in farm animals remains to be explored.

## 4. Livestock Exposure to PAEs and PAE Accumulation in Farm Animals

Data regarding animal exposure to environmental PAEs remains fragmented and limited to selected regions. Furthermore, a direct comparison of PAE concentrations in livestock from different areas is practically impossible. This is because, PAEs and their metabolites have been described in a wide variety of matrices, such as hair, urine, serum, muscle, internal organs, and eggs. Although comprehensive studies on livestock are limited, observations in other species indicate that each of these biological matrices reflects a distinct toxicokinetic profile and PAE exposure window, thereby complicating direct data synthesis. Levels of PAEs and their metabolites in urine, and serum samples reflect short-term exposure to these compounds [12,48]. In contrast, hair serves as a unique matrix, through which assessment of long-term, chronic, and retrospective exposure over weeks or months is possible, as PAEs become permanently incorporated into the keratin matrix during hair growth [49]. However, analyzing hair introduces significant analytical challenges, since a portion of the substances detected inside the hair may originate directly from the environment and do not reflect the levels of substances absorbed by the body [49]. Furthermore, comparing PAE concentrations across different animal species is also impossible, as their exact metabolism and kinetics in livestock remain unknown to date. In addition to these challenges, methodological variations, including sampling procedures, limits of detection and quantification, recovery tests, isotope-labeled internal standards, and laboratory contamination, must also be considered. This is particularly important because PAEs are highly susceptible to background contamination, as they may originate from plastic sampling containers, tubing, laboratory consumables, solvents, packaging materials, and the analytical environment itself [50]. Therefore, from a comparative standpoint, studies involving multiple matrices or samples from several regions analyzed under identical laboratory conditions appear to be the most valuable, whereas comparing results across different studies may be highly error-prone.

### 4.1. PAEs and Their Metabolites in Feces, Urine, Hair and Plasma

Since PAEs are rapidly metabolized in living organisms, studies on farm animal exposure to these compounds focus on determining the levels of their metabolites. A wide range of PAE metabolites—including phthalic acid (PA), monoethyl phthalate (MEP), mono(n-butyl) phthalate (MBP), monoisobutyl phthalate (MiBP), monobenzyl phthalate (MBzP), mono(2-ethylhexyl) phthalate (MEHP), mono(2-ethyl-5-hydroxyhexyl) phthalate (MEHHP), mono(2-ethyl-5-oxohexyl) phthalate (MEOHP), mono(5-carboxy-2-ethylpentyl) phthalate (MECPP), monoisononyl phthalate (MiNP), and mono(3-carboxypropyl) phthalate (MCPP)—has been detected in cow urine samples [51]. This study is highly valuable because it encompasses samples from cows across three distinct regions: the USA, India, and China. Generally, the highest occurrence frequency and concentrations were found in urine samples from cows in China, where all studied PAE metabolites were detected in over 90% of the samples. The highest levels were recorded for PA (median: 22 ng/mL), MBzP (median: 18 ng/mL), and MEHP (median: 9.7 ng/mL). Urine samples from cows in India contained significantly lower concentrations, despite a relatively high occurrence frequency ranging from 71.1% to 100%. In the Indian samples, the highest concentrations were recorded for MEP (median: 15 ng/mL), PA (median: 8.8 ng/mL), and MEHP (median: 7.9 ng/mL). The lowest concentrations were found in cow urine samples from the USA, where only five of the eleven substances were detected, with PA reaching the highest levels (median: 1.3 ng/mL) [51]. Such distinct differences between various regions are also known from observations regarding human exposure to PAEs [52,53], but their precise causes are not fully understood, much like the variations in PAE metabolite levels in the urine of cows included in the discussed study.

Interregional differences in PAE exposure may depend on a variety of factors (Figure 2). They may be fundamentally shaped by the degree of industrialization and urbanization, the core types of regional industry, and the density of wastewater treatment plants. In highly industrialized zones, elevated exposure is primarily linked to manufacturing emissions and urban runoff [53,54,55]. Agriculture can be a second important factor influencing the level of phthalates in the environment [1,4,54]. The increase in environmental phthalate levels is promoted by the extensive use of plastic mulch films, greenhouse sheeting, artificial fertilizers, plant protection products, and crop irrigation using recycled water. Furthermore, regional consumer practices—such as the widespread use of cosmetics, reliance on plastic packaging, and the total volume of municipal waste generated—heavily influence environmental contamination with PAEs [1,4]. Regional discrepancies in environmental protection legislation and plastic waste management may also be of great importance. Consequently, these complex socio-economic and infrastructural factors determine local PAE exposure profiles across different geographical regions. It can also be assumed that interregional differences in cattle exposure to PAEs may arise from field fertilization and irrigation practices, variations in feed production methods, and the types of packaging materials used.

PAE metabolites have also been described in hair samples collected from cows in Kyrgyzstan from areas located near Bishkek, the largest city in the country, which is characterized by a high degree of environmental pollution [56]. In that study, MEHP, monoisobutyl phthalate (MiBP), and MBP were detected in the highest percentage of samples (70.8%, 60.4%, and 60.4%, respectively). The highest concentrations were recorded for MEHP, ranging from below the limit of detection (LOD) to 128.3 μg/kg (median: 24.65 μg/kg). The medians for the remaining phthalates (MMP, MiBP, and MBP) did not exceed the limit of quantification (LOQ), while others (MEP and monocyclohexyl phthalate [MCHP]) were not detected in any of the samples [56].

Regarding PAEs, one previous study comprising two observations has described the presence of DEHP in sheep blood plasma. In the first observation, DEHP was detected in 34.7% of samples with a mean concentration of 450 ± 10 ng/mL; in the second, it was found in 42.6% of samples with a mean of 380 ± 20 ng/mL [57]. Furthermore, DEHP levels in sheep serum increase with age, and that pregnant ewes are particularly susceptible to DEHP exposure [57]. This may suggest that older and pregnant individuals are more sensitive to the effects of PAEs present in the environment. These results are consistent with observations in humans, where higher PAE levels in the elderly are attributed to age-dependent declines in metabolism and renal filtration, whereas in pregnant women, they are explained by intense physiological and hormonal changes characteristic of pregnancy [58,59].

The presence of PAEs has also been investigated in bovine feces; however, studies conducted in China did not confirm the presence of DMP, diethyl phthalate (DEP), DBP, BBP, DEHP, or DNOP in fecal samples collected from calves, pregnant cows, and milking cows [28]. The absence of PAEs in the analyzed fecal samples is somewhat surprising, given the ubiquitous presence of these compounds in the environment and the confirmed utility of fecal analysis for detecting PAEs in experimental animals [60]. The lack of PAEs in these samples may be due to the specific metabolism of these compounds in ruminants, which has not been studied so far, or the rapid biotransformation of PAEs into their metabolites. It may also result from the small number of samples included in the study, which originated from a single farm, or from methodological factors, such as the limit of detection. Therefore these findings should be interpreted cautiously because of the limited sample size.

### 4.2. PAEs in Raw Milk

PAE levels have been investigated in raw cow milk in Korea, Belgium, and Denmark. In Denmark, DEHP was present in raw milk at the highest concentrations, ranging from 7 μg/kg to 30 μg/kg, while the levels of other PAEs, such as DBP, BBP, DiNP, and diisodecyl phthalate (DiDP), were below the limit of detection [61]. In turn, research conducted in Korea has confirmed the presence of DBP, DEHP, and DMP in raw bovine milk. However, both their concentrations and frequency of occurrence were relatively low. DBP was detected in 67% of samples (mean: 30 μg/kg), DEHP in 50% (mean: 57 μg/kg), and DMP in 7% (mean: 1 μg/kg). The same study did not detect DEP, BBP, or DNOP [62].

The presence of PAEs was also confirmed in raw bovine milk samples collected in Belgium, where variations in PAE concentrations were observed between farms, seasons (winter vs. summer), and milking methods (manual vs. mechanical) [17,63]. Similar to the studies conducted in Korea and Denmark, the highest concentration was observed for DEHP, although distinct differences were noted between individual studies. In the first study, the highest concentration of DEHP was observed in machine-milked milk in winter, ranging from 75.6 μg/kg lipid to 161.9 μg/kg lipid (median: 123.5 μg/kg lipid), although the daily average in individual samples from selected farms could even reach 699 μg/kg lipid [17]. In the second study, a higher median concentration of DEHP was noted in summer (390 μg/kg lipid) than in winter (280 μg/kg lipid) [63]. Other PAEs, such as DiBP, DBP, BBP, and DNBP, were observed only at low concentrations in individual milk samples, whereas DMP, DEP, DCHP, and DNOP were not detected [17,63]. The PAE concentrations observed in milk from Belgium cannot be directly compared with those found in Korea and Denmark [62,63], as the authors presented concentrations in milk fat rather than in whole milk. Furthermore, these data are not highly representative because a small number of samples was analyzed (ranging from 1 to 14, depending on the milking method and season) and for this reason, their interpretation can be difficult. [17,63]. Nonetheless, the markedly higher concentrations of DEHP in milk fat [17,63] compared to whole milk [61,62] appear to support its lipophilic nature.

PAEs have also been investigated in sheep milk from Canada, where the mean levels of total PAEs ranged from 1930 ± 953 μg/kg to 3650 ± 2210 μg/kg, depending on the laboratory where the analyses were conducted [64]. However, that study was also limited by a small sample size of only five samples. Therefore, these results must be interpreted with great caution. PAE concentrations in sheep milk—comparing animals grazing on pastures fertilized with either sewage sludge or inorganic fertilizers—have also been described in the UK [65]. This study reported that the mean log concentration of PAEs in milk obtained from animals grazing on pastures fertilized with sewage sludge ranged from 2.32 μg/kg to 3.44 μg/kg, depending on grazing and milking time. These values did not statistically differ from the concentrations recorded in animals grazing on pastures treated with inorganic fertilizers (2.35–3.34 μg/kg) [65].

### 4.3. PAEs in Meat, Internal Organs and Fat

Similarly to raw milk, previous studies have reported the presence of PAEs in raw meat (muscle tissue) and internal organs, such as the liver, kidneys, heart, and lungs, which are frequently consumed by humans and are referred to as offal in the food industry. In sheep, according to Ikonomou et al. [64], the mean concentration of DEHP was 4060 ± 2530 μg/kg in muscle and 426 ± 242 μg/kg in the liver; however, that study was also limited by a small sample size of only five samples, which significantly limits the representativeness of the results. Other studies reported that the levels of PAEs in sheep muscle, liver, and perirenal fat depended not only on pasture contamination from sewage sludge but also on grazing duration [66,67,68]. In animals grazing on pastures fertilized with sewage sludge, the mean DEHP levels (depending on grazing duration) ranged from 63.0 μg/kg to 82.9 μg/kg in the muscle, and from 28.4 μg/kg to 73.4 μg/kg in the liver. In turn, the mean concentration of total PAEs in the perirenal fat ranged from 3.81 μg/kg to 3.97 μg/kg. For comparison, the values recorded in animals grazing on pastures treated with inorganic fertilizers were as follows: 40.5–97.5 μg/kg in the muscle, 23.3–73.7 μg/kg in the liver, and 3.67–3.99 μg/kg in the perirenal fat [66]. Moreover, the presence of DEHP has been demonstrated not only in adult sheep but also in fetal livers [67,68]. The mean log10 DEHP concentrations in the livers of fetuses were 0.445 mg/kg in animals from sewage-sludge-contaminated pastures and 0.182 mg/kg in the control group [67].

The presence of MEHHP, MEOHP, MBP, mono(n-octyl) phthalate (MNOP), MEHP, MBP, MEP, and monomethyl phthalate (MMP) was also studied in various porcine tissues: liver, heart, lung, kidney, muscle, stomach, pancreas, and spleen [19]. The highest concentrations of these substances were observed in the liver, muscle, and kidneys, while the lowest were found in the heart and stomach. Total concentrations of the studied substances ranged from 48.0 μg/kg in the heart to 108 μg/kg in the liver. MEHP was the dominant compound in all tested tissues, accounting for 30% to 57% of all mono-alkyl phthalate esters observed [19]. Similar observations regarding PAE metabolites in porcine tissues were reported by Yue et al. [69]. According to that study, total concentrations of mono-alkyl phthalate esters ranged from 9.9 ± 1.2 μg/kg in the heart to 94.0 ± 20.1 μg/kg in the liver. The highest concentrations were recorded for MBP (ranging from 6.1 ± 1.0 μg/kg in the heart to 55.1 ± 18.4 μg/kg in the liver) and MEHP (ranging from 3.8 ± 1.5 μg/kg in the heart to 26.4 ± 14.6 μg/kg in the liver). Yue et al. also described parent PAE levels in porcine tissues, where total mean concentrations ranged from 48.0 ± 6.0 μg/kg in the heart to 439.4 ± 49.3 μg/kg in the muscle. The most common PAEs were DiNP (from 9.4 ± 1.8 μg/kg in the heart to 72.0 ± 10.0 μg/kg in the muscle) and DEHP (from 18.8 ± 2.9 μg/kg in the heart to 24.5 ± 3.2 μg/kg in the muscle). The highest individual concentration levels were noted for di(n-decyl) phthalate (DnDP), reaching 280.7 ± 38.7 μg/kg in the muscle and 183.1 ± 193.7 μg/kg in the lungs. Interestingly, DnDP levels in all other tissues were below the limit of quantification [69].

Knowledge regarding PAE levels in the muscle and internal organs of other farm animal species is extremely limited. An average DBP concentration of 35.1 ± 0.2 μg/kg was observed in the muscle tissue of cattle collected immediately after slaughter, while dimethyl terephthalate (DMTP) and diisooctyl phthalate (DiOP) were absent [30]. In turn, DEHP was detected in chicken meat from Taiwan in 10% of samples (3 out of 30) at concentrations of 420 μg/kg, 430 μg/kg, and 450 μg/kg [70]. Simultaneously, the presence of BBP, DBP, DiDP, and DiOP was not observed in these samples. Recent investigations have identified the presence of DNOP, DEHP, and DBP in chicken adipose tissue, with concentrations reaching 83.5 ± 11.2 μg/kg, 62.6 ± 8.6 μg/kg, and 21.3 ± 1.7 μg/kg, respectively. Conversely, levels of DMP, DEP, and BBP remained below the limit of detection [71].

### 4.4. PAEs in Eggs

The first studies on PAE levels in eggs were conducted in the 1980s. In the albumen of control eggs obtained from Japanese markets, DBP concentrations ranged from below the LOD to 150 μg/kg, and DEHP concentrations from 50 to 40,000 μg/kg, whereas these compounds were not detected in the yolk [72]. Under experimental conditions, DEHP was also not detected in the egg yolk of control hens (without DEHP administration). Its levels in the egg albumen differed slightly from those found in market eggs, ranging from 10 to 410 μg/kg (mean: 210 μg/kg) or from 20 to 380 μg/kg (mean: 150 μg/kg), depending on the frequency of egg collection [72]. However, DEHP was detected only in the yolk of eggs (at concentrations up to 254,880 μg/kg) laid by hens receiving this substance orally [72]. This indicates that PAEs penetrate into eggs during their formation in the hen’s reproductive tract and confirms that the live exposure of hens to PAEs may affect the level of these compounds in eggs. More recent studies conducted in Turkey have confirmed the presence of PAEs in hen eggs and demonstrated a relationship between their concentrations and the type of poultry farming system [18]. Mean concentrations of total PAEs amounted to 21.1 μg/kg in battery eggs, 7.89 μg/kg in free-range eggs, and 11.7 μg/kg in organic eggs. The highest mean levels were recorded for DEHP (9.5 μg/kg in battery eggs, 3.5 μg/kg in free-range eggs, and 7.6 μg/kg in organic eggs), while the lowest levels were noted for BBP, which remained below the limit of quantification in all egg types [18].

Analyzing the above studies on the presence of PAEs in raw products of animal origin, distinct differences can be observed in the concentrations of PAEs and their metabolites, depending on the analyzed matrix and the region where the research was conducted. Despite these differences, the presence of DEHP and MEHP was most frequently observed, with their concentrations generally exceeding the levels of other PAEs and their metabolites. Higher levels were typically found in products with a higher lipid content, which may be attributed to the lipophilicity of PAEs. Clearly, existing studies regarding PAEs and their metabolites in raw animal products possess certain limitations, the most significant of which include a relatively low number of such studies and, in many cases, a limited number of analyzed samples. Furthermore, comparing these studies with one another is hindered by methodological differences, as well as variations in data analysis and presentation methods. Data regarding the levels of phthalates and their metabolites in farm animals and raw animal products are summarized in Table 1.

## 5. Post-Farm Contamination and Food Processing

This review aims to synthesize current knowledge regarding animal exposure to PAEs and their accumulation at the farm level, alongside the effects of these compounds on animal health. Nevertheless, the contamination of animal-origin products during the production phase remains a critical issue that cannot be overlooked. Therefore, although these aspects lie beyond the main scope of this paper and could themselves be the subject of a separate, extensive review, the key mechanisms related to them are briefly presented below.

In light of previous studies, it is well established that PAE levels in raw animal-origin products can differ significantly from those observed in processed products. This discrepancy arises because there are two primary sources of PAE contamination in animal products: in vivo exposure of livestock to environmental pollutants and technological contamination along the food production chain.

In the case of milk, the milking method can influence PAE concentrations, suggesting that milk can be contaminated with these compounds even prior to actual processing [17]. A significant increase in DEHP content, and to a lesser extent that of other PAEs, is observed during separation, pasteurization, and standardization, with the highest concentration levels found in milk powder, butter, and cheese [63,73,74]. The higher PAE levels in processed milk compared to raw milk likely result from contact with production machinery components that may contain these substances, such as sealants and tubing, during processing [75]. The increase in PAE concentrations in finished dairy products is driven by two factors. First, PAEs exhibit intensified and accelerated migration into food at higher temperatures, explaining the increased levels in pasteurized milk [76]. Second, due to their lipophilic nature, these compounds exhibit a higher affinity for matrices with a higher lipid content [43,45]. Earlier studies have also confirmed the migration of PAEs from packaging into dairy products [63].

Regarding meat, similar to milk, generally higher levels of PAEs have been found in processed meat compared to raw muscle tissue sampled immediately after slaughter. Available data indicate that even merely chilling meat and wrapping it in paper leads to an increase in PAE concentrations [30]. It has also been found that processing steps—such as chilling, grinding, deboning, trimming, and especially packaging—contribute to elevated PAE content in meat [30,76,77]. Moreover, subsequent studies have confirmed that a larger meat surface area in contact with the packaging (e.g., in the case of minced meat), as well as a higher lipid content, results in a higher degree of PAE contamination from the packaging [30,78].

Therefore, in light of the existing data, it can be inferred that processing stages and packaging migration may substantially contribute to PAE contamination of animal-derived foods.

## 6. Impact of PAEs and Their Metabolites on the Health of Farm Animals

### 6.1. Cows

#### 6.1.1. In Vitro Studies

The majority of in vitro studies concerning the effects of PAEs and their metabolites on cows have focused on the impact of MEHP on the female reproductive system. Existing studies have confirmed that MEHP may negatively affect oocytes, and this impact may be multidirectional. First, MEHP induces alterations in 456 genes in oocytes, including, among others, the *CYC1*, *MT-CO1*, *ATP5B*, *SOX2*, *DNMT3B*, *ASAH1*, *CCNA2*, and *POU5F1* genes [15,16,79,80]. The genes affected by MEHP are involved, among other processes, in the regulation of mitochondrial oxidative phosphorylation, the transcription process, cytoskeleton organization, metabolic pathways, apoptosis, cell cycle control, and pluripotency [15,16,80]. In oocytes exposed to MEHP, an increase in reactive oxygen species (ROS) levels and cellular organelle remodeling were also observed, involving a reduction in the number of category I mitochondria, type III cortical granules, and class I endoplasmic reticulum [79]. Furthermore, MEHP has been reported to affect oocyte nuclear maturation and DNA fragmentation [80], as well as significantly alter the proteomic profile of these cells [16]. These diverse effects exerted by MEHP lead to disruptions in oocyte development and maturation, as manifested by a higher percentage of oocytes remaining at the germinal vesicle stage, a reduction in the number of oocytes undergoing cleavage, a decrease in the percentage of developing blastocysts, and an increase in the proportion of apoptotic oocytes [15,80,81]. Similar adverse effects on oocyte maturation have also been demonstrated for DEHP, which exhibits adverse effects both on oocytes matured in the presence of this substance (direct effect) and on blastocysts developed from those oocytes (carryover effect), and may alter up to 290 genes in the blastocyst [16].

PAEs may affect not only oocytes but also other cell types within the female reproductive system, including luteal and endometrial cells [82,83]. In luteal cells treated with DEHP, disruptions in steroidogenesis were noted, manifesting as a marked decrease in progesterone levels [82]. Moreover, both DEHP and MEHP enhance the secretion of oxytocin from luteal and granulosa cells, without affecting cell viability [83]. In turn, studies on the effects of DEHP and MEHP on endometrial cells have shown that both substances cause an increase in the secretion of PGF2α, while simultaneously inhibiting PGE2 secretion, which leads to a distinct alteration in the PGF2α/PGE2 ratio [83].

The influence of PAEs on the male reproductive system is less understood. Nevertheless, previous studies have demonstrated that DEHP, MEHP, DBP, and BBP affect pluripotent stem cells isolated from testicular tissue [84,85]. This impact was manifested by enhanced apoptotic activity through a reduction in androgen receptor expression, a higher BAX/BCL-2 ratio, and an increase in the expression of the cyclin-dependent kinase inhibitor 1 (p21Cip1), alongside increased p53 transcriptional activity [84,85].

Knowledge regarding the impact of PAEs on other bovine internal organs and systems is also extremely limited. It has been found that a wide range of PAEs and their metabolites—such as DEP, di(n-propyl) phthalate (DPP), DBP, BBP, di(n-pentyl) phthalate (DnPP), di(n-hexyl) phthalate (DHP), DCHP, DEHP, MBP, and PA—affect calcium signaling via nicotinic acetylcholine receptors (nAChR) in bovine adrenal chromaffin cells through the inhibition of the nAChR-coupled Ca^2+^ response [86,87,88]. Notably, the most significant alterations were observed under the effects of DPP, BBP, and DBP, which were ten times more potent in affecting nAChRs than estradiol [88]. Moreover, DBP and MBP inhibit the activity of superoxide dismutase (SOD), an antioxidant enzyme isolated from bovine lungs, probably through binding to the enzyme’s active site and forming hydrogen bonds with the R143 residue, which is crucial for binding ROS [89]. Another study has shown that DEHP exhibits the ability to bind to the A-T-rich regions of the DNA minor groove isolated from calf thymus cells, inducing a decrease in base stacking and an increase in right-handed helicity, without significant DNA damage [90].

#### 6.1.2. In Vivo Studies

In vivo investigations into the effects of PAEs in cows are much less numerous. However, it has been shown that administering water containing DEHP to cows for three days impaired follicular development, which manifested as a smaller diameter and reduced growth rate of ovarian follicles in comparison to control animals [91]. Moreover, DEHP administration led to decreased estradiol concentrations and a reduced corpus luteum volume. Follicular fluid collected from DEHP-treated animals contained MEHP, and exposure to this fluid inhibited the maturation of cultured bovine oocytes [91].

### 6.2. Goats

#### 6.2.1. In Vitro Studies

The knowledge concerning the influence of PAEs on cells isolated from goats is extremely limited and restricted to only two investigations. In mammary gland epithelial cells, DEHP-induced changes manifest as mitochondrial and endoplasmic reticulum damage, elevated oxidative stress, and the accumulation of ROS via mitogen-activated protein kinase (MAPK), ribosomal, ferroptosis, and p53 signaling pathways [92]. Furthermore, DEHP in these cells induces alterations in 113 genes, including *GCLC*, *SQSTM1*, and *ATF3*, which are involved in the regulation of cell proliferation, apoptosis, and oxidative stress [92]. The second study has shown that MEHP distinctly influences Sertoli cells isolated from immature goat testes, leading to an increased degree of vacuolization, the appearance of nuclear membrane ruptures, and mitochondrial alterations, such as swelling and rupture [93]. These changes were dependent on both exposure time and MEHP concentration, with low concentrations triggering apoptosis, while high concentrations led to cell necrosis [93].

#### 6.2.2. In Vivo Studies

In vivo studies on the effects of PAEs on goats are also limited, but important from the standpoint of their environmental exposure to these substances. These studies investigate the impact of a mixture of PAEs administered via feed, reflecting the environmental situation where animals are often exposed to the simultaneous action of multiple toxic compounds exhibiting synergistic effects [94,95].

A mixture of DEP, DiBP, and DPP given orally to pregnant Bengal goats caused a decrease in serum levels of sodium, chloride, and calcium, as well as certain hormones, including estrogen, progesterone, luteinizing hormone, and thyroxine [94]. These changes were accompanied by a marked impact on postpartum reproductive traits, including prolonged gestation, an increased risk of abortion, delayed postpartum estrus, and retained placenta [94]. Oral exposure of goats to a mixture of PAEs resulted in a significant decline in physiological parameters, such as body condition score, respiration rate, and heart rate, as well as changes in hematological parameters. Namely, in treated animals, decreases in RBC count, WBC count, hemoglobin concentration, hematocrit, RBC indices, and lymphocyte count were noted, alongside a simultaneous increase in neutrophil count [95]. A mixture of PAEs also reduced serum levels of glucose, total protein, albumin, and total cholesterol, while increasing the activities of aspartate aminotransferase and alanine aminotransferase, as well as blood urea nitrogen levels [95].

### 6.3. Horses

#### In Vitro Studies

To the best of the author’s knowledge, only one previous study has addressed the influence of PAEs on horses. This study showed that high doses of DEHP induced apoptosis while simultaneously increasing ROS levels in oocytes and inhibiting their maturation. However, this impact did not alter the ooplasmic energy status of matured oocytes, which retained their fertilization potential [96].

### 6.4. Pigs

#### 6.4.1. In Vitro Studies

Studies on the toxic activity of PAEs in pigs primarily focus on the female reproductive system, especially on ovarian cells. MBP induces abnormal epigenetic modifications in oocytes, affecting protein expression (e.g., H3K36me3 and SETD2), increasing DNA breakage, and intensifying apoptosis. Furthermore, it affects genes leading to impaired meiosis, mitochondrial dysfunction, and disorders in early embryonic development in these cells [97,98]. A similar effect on oocytes, involving meiotic disruption, impaired mitochondrial function and integrity, and alterations in spindle conformation and chromosome alignment, has been reported for BBP [99]. A particularly pronounced negative effect on oocytes—involving the suppression of meiosis, cumulus expansion, and progesterone synthesis in granulosa cells—has been reported for a mixture of BBP and other endocrine-disrupting chemicals, such as bisphenol A and 4-chloro-3-methylphenol [100]. BBP also impairs cumulus expansion and inhibits pre-implantation embryo development, which occurs at both the gene expression and metabolic levels, resulting in a reduction in the number of embryos reaching key developmental stages [101]. Moreover, BBP impairs embryonic development by affecting the endoplasmic reticulum, Golgi apparatus, and mitochondrial membranes, as well as inducing oxidative stress and apoptosis [102]. Other substances that affect the cells of the female reproductive system include DEHP and its metabolite MEHP. Furthermore, DEHP influences polar body extrusion in porcine oocytes [103] and progesterone production by oocyte–cumulus complexes [101]. In turn, MEHP may disrupt meiosis, induce apoptosis and oxidative stress, as well as affect DNA methylation levels in oocytes [104]. Interestingly, studies on the effects of PAEs on the porcine reproductive system focused not only on substances with known potent toxic properties (DEHP, BBP), but also on compounds introduced into the industry as their substitutes, such as DiNP and DiDP, which were considered less harmful to living organisms. These studies demonstrated that these substitute compounds also negatively affected reproductive cells in a manner similar to the substances being phased out of the industry due to their high toxicity. Namely, it has been shown that DiNP inhibits meiosis, causes mitochondrial dysfunction, intensifies oxidative stress, and induces apoptosis in oocytes, as well as reduces fertilization capacity by disrupting the dynamics of sperm binding and fusion proteins [105]. Moreover, DiNP may affect granulosa cell hormone production, but the results of previous studies remain unclear. Some studies report that DiNP inhibits estradiol production within primary granulosa cells while simultaneously increasing progesterone synthesis [106], whereas others show a DiNP-induced increase in estrogen production with a simultaneous inhibition of progesterone synthesis, alongside a disruption of the redox balance via elevated free radical levels in these cells [107]. An effect on the hormonal activity of granulosa cells was also demonstrated for DiDP and DNOP [106].

Information regarding the effects of PAEs on cells isolated from porcine internal organs and systems other than the female reproductive system is extremely limited. To the best of the author’s knowledge, only two previous studies have addressed this subject. One of them showed that DBP induces apoptosis in testicular cells [108]. The second study found that DEHP decreases the viability of aortic valve endothelial cells and triggers apoptosis via the activation of apoptotic pathway-related genes, such as *Bax*, *Caspase-3*, and *Caspase-9*, alongside the simultaneous suppression of *Bcl-2* [109].

#### 6.4.2. In Vivo Studies

Previous research has examined the effects of orally administered DEHP on the reproductive system of newborn piglets and prepubertal animals. In males, DEHP caused a decrease in progesterone and pregnenolone levels and affected germ cells by limiting their migration and increasing their mortality, while in females, this substance increased progesterone levels but did not alter the number of ovarian follicles, nor did it induce apoptosis or changes in the gene expression of steroidogenic enzymes [110]. However, other studies did not confirm that oral exposure of young boars to DEHP causes significant changes in the structure of the reproductive system, either immediately after exposure or upon reaching sexual maturity. Specifically, the number of Sertoli cells, the percentage area of Leydig cells, and the incidence of germinal epithelial lesions were similar between the control and DEHP-treated groups, and the only observed change was the DEHP-induced precocious maturity of the bulbourethral glands [111]. Furthermore, oral administration of DEHP to young males was not shown to affect the mating behavior of adult animals, despite its demonstrated impact on the hypothalamic–pituitary–gonadal axis [112]. However, it should be emphasized that the impact of orally administered DEHP on the male reproductive system remains incomplete. Some studies found no effect of DEHP administered to young animals on sperm count or the penetration rate of adult boars, nor on sperm plasma membrane stability, acrosomal integrity, chromatin structure, or DNA denaturation [113,114]. In turn, other observations have confirmed that exposure of young boars to DEHP may lead to changes in the semen of adult animals. These changes included a significant decrease in total sperm motility, a lower percentage of linearly motile spermatozoa, and a larger amplitude of lateral head displacement, but interestingly, a lower percentage of spermatozoa with tailless or defective heads compared to the control group [115,116]. Some changes in the male reproductive system—consisting of an increase in serum testosterone levels and a larger Leydig cell area in the testes—have also been observed after intramuscular administration of DEHP, which may suggest that early-life exposure to DEHP can have delayed effects on the male reproductive system in adulthood [117].

### 6.5. Sheep

#### 6.5.1. In Vitro Studies

The only study on the influence of DEHP on sheep cells confirmed that this substance affects mitochondria and causes an increase in ROS levels within oocytes, with these changes being more pronounced when oocytes are incubated simultaneously with DEHP and cadmium [118].

#### 6.5.2. In Vivo Studies

In sheep, in vivo studies on the effects of PAEs are more numerous than in vitro studies. These studies have shown that intravenously and intramuscularly administered DEHP shortens the estrous cycle duration and reduces corpus luteum size, with a simultaneous increase in serum progesterone and estradiol concentrations, alongside decreased LH levels. This suggests that DEHP not only reduces the size and lifespan of corpora lutea but also alters steroid metabolism [119,120]. On the other hand, no effect of DEHP exposure was found on preovulatory follicular dynamics or ovulatory efficiency [120].

An interesting in vivo experiment simulating the natural exposure of sheep to harmful substances present in the environment was conducted by Ghasemzadeh-Hasankolaei et al. [121]. They studied the impact of sheep grazing on pastures treated with biosolids containing a mixture of endocrine-disrupting chemicals (including phthalates) on their adult offspring. It was found that the body weight of offspring from the exposed ewes was significantly lower than that of the control animals. Moreover, exposure to these endocrine-disrupting chemicals caused changes in gene expression (such as *LEPR*, *NPY*, *ESR1*, *DNMT1*, and *DNMT3A*) in the hypothalamus, alongside disruptions in lipid metabolism gene expression in the visceral adipose tissue—manifested by a decrease in the mRNA expression levels of lipogenic markers (*ZNF423* and *LPL*)—and in the subcutaneous fat, which showed an increase in *DLK1* mRNA expression [121].

### 6.6. Poultry

#### 6.6.1. In Vitro Studies

While studies on the effects of PAEs on cells isolated from various poultry organs are relatively numerous, they focus exclusively on DEHP. DEHP-induced apoptosis and the intensification of oxidative stress have been described in chicken cardiomyocytes, accompanied by an increase in the mRNA and protein expression levels of *Caspase-3*, *Caspase-9*, *Cyt c*, and *Bax* [122]. In turn, necroptosis in cardiomyocytes, which was also noted under the influence of DEHP, is connected with increased cytosolic Ca^2+^ levels resulting from the upregulation of gene expression within the necroptosis-calcium pathway, including *RIPK1*, *RIPK3*, *MLKL*, *FAS*, *Caspase-8*, *CAMKII*, and *SERCA* [123]. In addition, the impact of DEHP on cardiomyocytes is characterized by cellular hypertrophy, which is accompanied by the upregulation of the mRNA and protein expression of atrial natriuretic peptide, natriuretic peptide A, and β-myosin heavy chain (β-MHC), as well as changes in mitochondrial function and structure [124]. The former effect is associated with disrupted glucose metabolism, as evidenced by the upregulation of the mRNA expression of related genes, including *GLUT1*, *GLUT2*, *GLUT3*, *GLUT8*, and several insulin-like growth factor-binding proteins (*IGFBP1–5*). Conversely, the latter effect results from the DEHP-induced downregulation of the expression of mitochondrial function-related transcription factors, such as *PPARα*, *PPARγ*, *PGC-1α*, *NRF1*, *NRF2*, and *TFAM* [124].

Previous studies have also indicated that DEHP activates the ROS/TLR4/MyD88 pathway, a key signaling pathway involved in the regulation of inflammation, the immune response, and oxidative stress in macrophages and lymphocytes [125]. This activation leads to M1 polarization in macrophages, as well as pyroptosis and necroptosis in lymphocytes. DEHP-induced necroptosis resulting from disturbances in the MAPK/NF-κB signaling pathway—which was accompanied by an increase in ROS levels leading to decreased cell viability—has also been observed in lymphoblasts [126].

#### 6.6.2. In Vivo Studies

In vivo studies on the impact of PAEs on poultry health date back to the 1980s, when it was reported that oral administration of DEHP causes diarrhea, anorexia, growth inhibition, and a decrease in egg production [72,127], as well as affects lipid metabolism, leading to lower lipid levels in the blood serum and liver [127]. The toxic effects of DEHP on poultry have been confirmed by more recent studies. These studies not only confirmed the negative impact of PAEs—either alone or in combination with other microplastic components—on food intake, body weight gain, the gut microbiota, and lipid metabolism [125,128,129], but also demonstrated their detrimental effects on specific internal organs [125,126,128,130].

Orally administered DEHP caused abnormal spleen enlargement in chickens, accompanied by histopathological changes—such as the blurring of the border between the red and white pulp, a decrease in white pulp volume, a drop in the lymphocyte count, fragmentation of lymphocyte nuclei, the presence of apoptotic vesicles, and the presence of numerous inflammatory cells [125]. These changes were caused by DEHP-induced apoptosis and necroptosis via the ROS/TLR4/MyD88 pathway, alongside macrophage M1 polarization involved in inflammatory processes [125]. Similar histopathological alterations under the influence of DEHP were also reported in the bursa of Fabricius, where a blurred cortex–medulla boundary, significantly fewer lymphocytes in the medulla, and nuclear condensation, fragmentation, or lysis in lymphocytes were noted [126]. Moreover, the same regulatory pathway found in the spleen—activated by DEHP and leading to programmed necrosis and pyroptosis—was active in the chicken duodenum, where DEHP administration caused a reduction in crypt depth and villus length [130]. Previous studies have also confirmed the negative impact of DEHP on the reproductive system of laying hens, manifested by ovarian follicle atresia, atrophy of the uterine epithelium, changes in egg yolk color, and reduced shell thickness [128]. Investigations into the cellular mechanisms of these changes revealed that DEHP disrupts ovarian pathways—such as neuroactive ligand-receptor interactions, as well as glutathione and glycerophospholipid metabolism—affects genes related to the extracellular matrix, ion transport, and eggshell mineralization in the uterus, and activates apoptotic pathways [128]. DEHP also triggers apoptosis in hepatocytes by increasing the mRNA and protein levels of apoptosis-related genes (*p53*, *Caspase-9*, *Caspase-3*, and *Bax*), as well as the levels of apoptotic protein markers, including cleaved Caspase-9 and cleaved Caspase-3 [131]. Furthermore, it exacerbates oxidative stress in hepatocytes and activates ferroptosis—a type of non-apoptotic cell death dependent on changes in myo-inositol oxygenase (MIOX) activity and the Signal Transducer and Activator of Transcription 3 (STAT3) signaling pathway [131,132].

#### 6.6.3. In Ovo Studies

An important part of research on PAEs in poultry involves in vivo studies evaluating the impact of these substances on the chicken embryo. The first studies of this type, conducted in the 1970s, showed that exposure to PAEs during embryonic development results in increased mortality and congenital malformations—including crania bifida, anophthalmia, and nervous system abnormalities—in newly hatched chicks [133]. More recent studies have shown that DEHP and DBP reduce the hatching percentage and increase the number of late hatchings, while also causing teratogenic changes, such as the opening or weakening of abdominal muscles leading to omphalocele or gastroschisis [134]. Moreover, DEHP damages chicken DNA and exhibits neurotoxic activity, resulting in the abolishment of imprinting performance [134]. The embryotoxic effects of PAEs and their metabolites in chickens result from increased oxidative stress and apoptosis, although to date, no adverse effects of PAEs on the development of the embryonic reproductive system have been confirmed [135,136].

### 6.7. Summarization of Studies on PAEs Impact on the Livestock and Their Environmental Relevance

**Figure 3:**
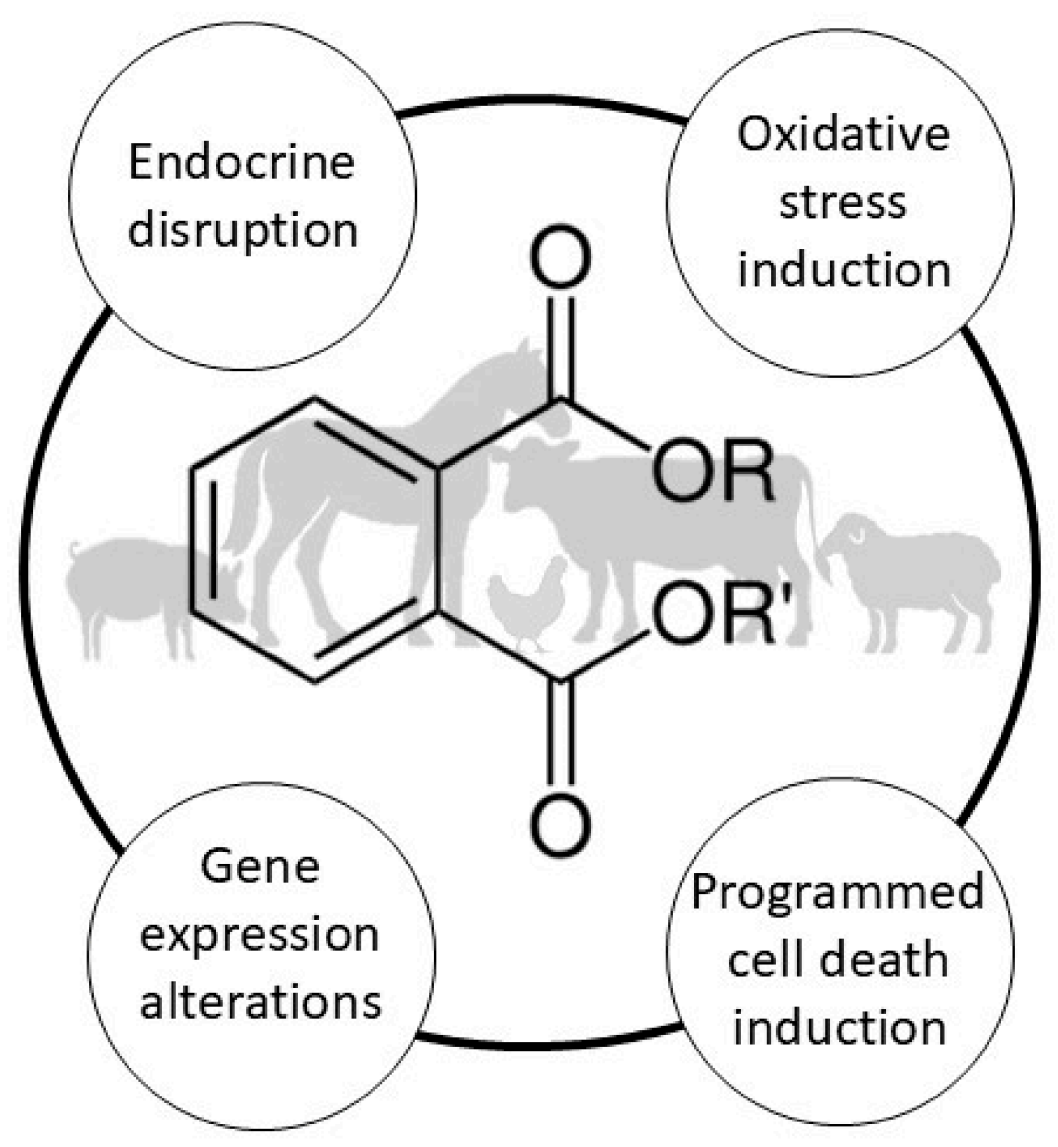
The main toxic mechanisms of action of PAEs in livestock.

Previous studies on the effects of PAEs on livestock indicate that these compounds can have multidirectional effects. Based on these findings, several main mechanisms of PAE action in livestock can be distinguished (Figure 3), which are similar across all farm animal species. These mechanisms, also known from observations in humans and experimental animals, include: (1) endocrine disruption; (2) induction of oxidative stress reactions; (3) alteration of gene regulation and expression (including epigenetic modifications); and (4) induction of programmed cell death (apoptosis, pyroptosis, and/or necroptosis).

Most existing studies on the effects of PAEs on livestock have focused on DEHP and its metabolite MEHP, compounds whose toxic effects are relatively well-documented in other living organisms as well. A significantly smaller number of publications focus on other PAEs, such as BBP, DPP, and DBP. Furthermore, there are also studies—albeit isolated ones—demonstrating adverse effects on livestock from phthalates considered safer alternatives to DEHP in the industry, which were originally believed to be less toxic, such as DiNP and DiDP [105,106]. The impact of these substances on the porcine reproductive system may suggest that these compounds are not as safe as assumed when they were introduced into the industry as replacements for DEHP. This is also confirmed by contemporary studies on experimental animals describing the diverse toxic effects of DiNP and DiDP [137,138]. However, it should be underlined that the evidence concerning DPHP, DiNP and several newer replacement plasticizers in livestock remains extremely limited, and their toxicokinetics and biological effects under realistic exposure conditions require further investigation.

The vast majority of studies on PAE toxicity in livestock focus on the reproductive system. The multidirectional adverse effects of these compounds on both the female and male reproductive systems, as well as embryonic development, may suggest that PAEs play role in livestock reproduction and, consequently, represent a factor that adversely affects animal breeding. However, it should be noted that in vitro and in ovo studies, which dominate the existing research on the effects of PAEs on livestock, do not reflect real environmental exposure in animals. In such studies, it is difficult to determine whether the tested dose corresponds even approximately to environmental exposure, since cells are directly subjected to the action of the tested substance without considering the route of exposure, the metabolic transformations of the substance in the body, or the regulatory influence of the nervous and/or endocrine systems. Therefore, it seems that in vivo studies better reflect the environmental exposure of livestock to PAEs. However, even this type of study usually does not reflect environmental exposure, as the duration of the experiment, the concentration of the substance administered to the animal, and/or the route of administration usually do not correspond to “natural” conditions. Animals are exposed to PAEs long-term, virtually from fetal life, and the routes of exposure vary (including the digestive system, respiratory system, and skin), making it very difficult to accurately replicate these factors under laboratory conditions. Moreover, due to limited studies on PAE concentrations in feed, water, and the environment of livestock buildings, determining the environmental dose of PAEs to which animals are exposed is extremely challenging. Another important factor usually overlooked in animal experiments is that the organism is simultaneously exposed to multiple harmful substances contained in the environment, which often exhibit synergistic effects. In such cases, on the one hand, the toxic action of the tested substances may be intensified, while on the other hand, even a lower dose of the tested compound may induce toxic effects. Conclusions drawn from such experimental studies, where the investigated PAE dose is typically distinctly higher than its environmental concentration, should not be presented as direct evidence of effects occurring at typical field-exposure concentrations. Therefore, the adverse effects of PAEs on the organism described in the literature can, at most, suggest a negative impact of these compounds on animal husbandry, but they do not definitively confirm this fact. In vivo studies determining the effects of mixtures of endocrine-disrupting compounds—which, unfortunately, remain highly limited—appear to carry the greatest environmental significance [94,95,121]. In particular, studies simulating environmental exposure by grazing animals on pastures treated with biosolids containing a mixture of endocrine-disrupting chemicals (including PAEs) best reflect real-world conditions in animal husbandry [121]. The adverse effects of PAEs on livestock observed in this type of study suggest that these compounds can negatively impact animal health, thereby holding implications for livestock production.

An overview of previous research regarding the effects of PAEs and their metabolites on livestock is provided in Table 2.

## 7. Knowledge Gaps and Future Perspectives

Current knowledge regarding PAE exposure in livestock is highly fragmented, and clarifying all aspects of this issue requires further multidirectional research. The primary direction of such research should be to determine the sources of livestock exposure within the farm environment. Although existing studies address the presence of PAEs in animal feed and drinking water, they are scarce and have been conducted on a highly limited number of samples, thereby failing to sufficiently identify the hazards associated with this issue. Furthermore, the limited sample size means that the results of these studies should be interpreted cautiously. Moreover, to date, there are no studies investigating PAE concentrations in dust or bedding within animal housing, on pastures, or in the ambient air. There is also a lack of research regarding the relationship between PAE exposure and husbandry, maintenance, or veterinary practices. Comprehensive research into the sources of livestock exposure to these compounds is essential to determine their actual significance in animal husbandry.

The second direction of research involves assessing PAE and metabolite levels in the animal organism. Studies that link PAE concentrations in the farm environment with the levels of these compounds in various biological matrices—such as serum, urine, hair, milk, and feces—appear particularly valuable. To date, such studies are virtually non-existent; the levels of phthalates or their metabolites have been described only in isolated, disparate matrices using relatively few samples, which hinders the direct comparison of results. It is also highly critical to understand how environmental PAE concentrations translate into levels of these compounds and their metabolites in livestock—a process that is fundamentally governed by the toxicokinetics of both the parent PAEs and their metabolites. Regrettably, a major limitation in the current literature is that the toxicokinetics of PAEs in livestock species remains virtually unknown. Data regarding PAE absorption rates, metabolic pathways, metabolic rates, and the distribution of PAEs and their metabolites across various tissues are critically limited. Addressing these research gaps through standardized analytical protocols and livestock kinetic studies represents one of the most critical objectives for future research on PAEs in farm animals. This task is further complicated by the likelihood that PAE toxicokinetics varies significantly across different livestock species, such as cattle, swine, poultry, and horses. These variations are expected to depend heavily on gastrointestinal anatomy, metabolic rates, endocrine activity, and numerous other species-specific physiological factors.

The third critical avenue for future research is to determine the impact of environmental PAE exposure on the health and productivity of livestock. Although existing studies on this topic primarily focus on experimental conditions and, in most cases, do not reflect real-world environmental exposure. It seems imperative to conduct environmental, toxicological, and clinical studies that delineate the relationships between the degree of PAE exposure, the concentration of these substances and/or their metabolites in the body, and the clinical status and productivity of livestock.

Another critically important direction for future research is the precise determination of PAE and metabolite concentrations in raw products of animal origin, such as milk, meat, eggs, and wool. Current knowledge on this subject is highly limited, in contrast to studies focusing on the contamination of processed food during production and packaging. For instance, although studies on PAE content in raw milk exist, there are no observations regarding the levels of PAE metabolites in such matrices. Given the rapid metabolism of these compounds, such studies appear highly necessary and intriguing. This type of research is essential to determine whether raw animal products can be contaminated with PAEs as a result of lifetime animal exposure, and whether they may consequently pose a risk to consumers. Importantly, comprehensive studies regarding the levels of PAEs and their metabolites in livestock and animal-origin products should ideally be performed within a single laboratory or be precisely standardized and reproducible. Although this is highly challenging, such an approach would eliminate methodological differences related to the equipment used, detection limits, and reagents, thereby enabling the direct comparison of results from different regions of the world.

Only multidirectional, integrated research addressing all of the aforementioned areas will allow for a comprehensive understanding of all aspects related to phthalates in livestock. This is particularly vital within the framework of the One Health concept. From this perspective, investigating the interrelationships between environmental phthalate levels, livestock exposure, animal health, and phthalate levels in raw animal products—and consequently consumer exposure and human health—is of utmost importance. Developing this knowledge will enhance environmental monitoring, allow for a robust risk assessment of food safety, and effectively inhibit the transfer of phthalates within the environment–animal–human triad.

## 8. Conclusions

Summarizing the research to date on livestock exposure to PAEs, the exposure levels clearly depend on multiple factors. The most significant among these factors appear to be the animal species and the geographical region where the study was conducted. Furthermore, the observed levels of PAEs and their metabolites are dependent on the type of matrix analyzed. Other factors influencing livestock exposure include husbandry practices, feed types, and pasture fertilization methods. However, it must be emphasized that current knowledge regarding PAE exposure in livestock remains relatively limited, and numerous associated aspects require further comprehensive investigation. Some of the existing studies on livestock exposure to PAEs were conducted with a very limited sample size, meaning that their results should be interpreted with great caution.

Previous studies have also shown that environmental PAEs, to which livestock are exposed, can migrate into animal-derived raw products consumed by humans, such as milk, meat, offal, and eggs. Although in vivo exposure of animals results in lower PAE levels than subsequent industrial processing, it may still contribute to increased human exposure to these substances.

Existing studies suggest that PAEs can negatively affect livestock health. The impact of DEHP on the reproductive system is the best-documented in this regard. Exposure to this compound can result in reproductive disorders, leading to impaired fertility and consequently adversely affecting animal breeding. The effects of PAEs on other organs and internal systems of livestock are much less understood. However, it should be noted that most studies to date have been conducted under experimental conditions, often utilizing PAE doses much higher than those observed in the environment. Therefore, the results of such studies do not provide direct evidence of adverse health effects in livestock at concentrations typical of field-exposure conditions. The impact of environmental livestock exposure to PAEs on health status remains poorly understood.

Available evidence indicates that PAE exposure represents a potential concern for livestock health and food safety, although the magnitude of these risks under realistic field exposure conditions remains insufficiently characterized.

## Figures and Tables

**Table 1:** Summary of current knowledge on PAEs and their metabolites in livestock. The units are µg/kg for solid matrices, eggs and milk (except for study marked with ^(A)^, where the unit is µg/kg fat) and ng/mL for other liquid matrices.

Country	Matrix	n	Concentration Levels of Phthalates and Their Metabolites	References
**PHTHALATES**				
**COWS**				
China	feces	27	DMP (ND), DEP (ND), DBP (ND), BBP (ND), DEHP (ND), DNOP (ND)	[28]
Belgium	raw milk	10	Winter: DMP (ND), DEP (ND), DiBP (ND-52, median: 23); DBP (ND-<LOQ), BBP (<LOQ-21, median: <LOQ), DEHP (201–500, median: 280) DCHP (ND), DNOP (ND) Summer: DMP (ND), DEP (ND), DiBP (nd-<LOQ), DBP (nd-15, median: ND), BBP (ND-16, median: ND), DEHP (ND-788, median: 390), DCHP (ND), DNOP (ND)	[63] ^(A)^
		14	Milked by hand: DMP (ND), DEP (ND), DiBP (ND-123), DBP (ND-25.2), BBP (ND-37.2), DEHP (ND-91), DCHP (ND), DNOP (ND)	[17] ^(A)^
		5	Milked by machine: DMP (ND), DEP (ND), DiBP (ND-21.5), DNBP (ND-<LOQ), BBP (ND-44.6), DEHP (ND-161.9), DCHP (ND), DNOP (ND)	
Denmark		18	DBP (<LOD) BBP (<LOD) DEHP (7–30), DiNP (<LOD), DiDP (<LOD)	[61]
Korea		30	DMP (ND-17, mean: 1), DEP (ND), DBP (ND-99, mean: 30), BBP (ND), DEHP (ND-154, mean: 57), DNOP (ND)	[62]
Russia	muscles	40	DMTP (ND), DBP (mean 35.1 ± 0.2), DiOP (ND)	[30]
**SHEEP**				
Spain	blood plasma	150 101	DEHP (50–2810, median: 450 ± 10) DEHP (210–570, median: 380 ± 20)	[57]
Canada	raw milk	5	Total concentration of PAEs (mean: 1930 ± 953 or 3650 ± 2210, depending on the laboratory, where analysis was performed)	[64]
	liver	5	DEHP (mean: 426 ± 242)	
	muscle	5	DEHP (mean: 4060 ± 2530)	
UK	raw milk	149	Total concentration of PAEs (Mean log10) on sewage sludge-treated pastures: from 2.32 to 3.44 * on pastures treated with inorganic fertilizers: from 2.35 to 3.34 *	[65]
	liver	89	DEHP (Mean log10) on sewage sludge-treated pastures: from 28.4 to 73.4 ** on pastures treated with inorganic fertilizers: from 23.3 to 40.3 **	[66]
	muscles		DEHP (Mean log10) on sewage sludge-treated pastures: from 63.0 ± 3.969 to 82.9 ** on pastures treated with inorganic fertilizers: from 40.5 ± 1.640 to 97.5 **	
	kidney fat		Total concentration of PAEs (Mean log10) on sewage sludge-treated pastures: from 3.81 to 3.97 ** on pastures treated with inorganic fertilizers: from 3.67 to 3.99 **	
	liver	21	DEHP (mean log10) on sewage sludge-treated pastures: 0.390; on pastures treated with inorganic fertilizers: 0.265	[67]
	fetal liver	28	DEHP (mean log10) on sewage sludge-treated pastures: 0.445; on pastures treated with inorganic fertilizers:0.182	
	maternal and fetal liver	72	DEHP has been observed, but the authors do not provide exact numerical concentrations, presenting them only in a graph, which makes it impossible to state the precise values. As shown in the graphs, mean log10 of DEHP concentrations fluctuate around 2.5–3 both in maternal and fetal liver	[68]
**PIGS**				
China	liver	16	DNOP (0.6–1.3, mean 0.9 ± 0.2), DnDP (<LOQ), DBP (8.3–14.5, mean: 11.7 ± 2.0), DEP (1.3–4.5, mean: 2.6 ± 0.9), DMP (0.8–4.8, mean: 2.3 ± 1.0), DiBP (8.8–18.1, mean: 12.2 ± 2.2), DHP (16.5–35.9, mean: 22.8 ± 5.2), DEHP (5.7–12.9, mean: 7.9 ± 2.0), DCHP (<LOQ); BMPP (<LOQ)	[69]
	lung		DNOP (<LOQ), DnDP (<LOQ-638.54, mean: 183.07 ± 193.74), DBP (9.3–18.8, mean: 13.9 ± 2.6), DEP (6.6–8.3, mean: 7.2 ± 0.5), DMP (1.8–6.4, mean: 3.0 ± 1.3), DiBP (12.8–25.0, mean: 17.9 ± 3.5), DHP (17.2–28.1, mean: 22.1 ± 3.0), DEHP (12.7–17.0, mean: 14.6 ± 1.2), DCHP (16.5–27.8, mean: 21.6 ± 3.1), BMPP (11.8–21.3, mean: 16.3 ± 2.6)	
	muscle		DNOP (<LOQ), DnDP (202.37–348.92, mean: 280.7 ± 38.74), DBP (92.1–187.9, mean: 132.8 ± 23.3), DEP (5.0–5.9, mean: 5.4 ± 0.2), DMP (2.6–8.8, mean: 5.7 ± 1.9), DiBP (56.8–88.7, mean: 72.0 ± 10.0), DHP (20.8–32.8, mean: 24.5 ± 3.2), DEHP (14.1–19.7, mean: 15.6 ± 1.4), DCHP (20.0–52.5, mean: 25.2 ± 7.5), BMPP (96.6–200.7, mean: 138.1 ± 23.5)	
	spleen		DNOP (<LOQ), DnDP (<LOQ), DBP (17.0–83.8, mean: 48.3 ± 22.9), DEP (28.5–65.6, mean: 45.5 ± 12), DMP (0.9–18.1, mean: 5.8 ± 4.5), DiBP (15.1–53.4, mean: 27.0 ± 9.7), DHP (15.8–35.7, mean: 22.7 ± 5.5), DEHP (12.4–19.5, mean: 15.0 ± 2.0), DCHP (<LOQ), BMPP (<LOQ)	
	kidney		DNOP (<LOQ), DnDP (<LOQ), DBP (34.8–95.0, mean: 66.5 ± 21.0), DEP (21.5–45.0, mean: 31.2 ± 8.1), DMP (1.4–7.7, mean: 3.4 ± 2.0), DiBP (21.5–48.9, mean: 31.8 ± 8.2), DHP (14.3–37.1, mean: 17.5 ± 5.3), DEHP (11.7–20.8, mean: 13.1 ± 2.1), DCHP (5.2–10.3, mean: 7.9 ± 1.5), BMPP (<LOQ)	
	heart		DNOP (<LOQ), DnDP (<LOQ), DBP (7.3–14.3, mean: 10.7 ± 1.9), DEP (0.4–1.6, mean: 1.1 ± 0.3), DMP (0.5–1.8, mean: 1.4 ± 0.3), DiBP (5.8–12.4, mean: 9.4 ± 1.8), DHP (13.8–23.1, mean: 18.8 ± 2.9), DEHP (4.5–10.3, mean: 6.6 ± 1.5), DCHP (<LOQ); BMPP (<LOQ)	
**CHICKEN**				
Taiwan	muscles	30	DEHP (420–450), BBP (ND), DBP (ND), DiDP (ND), DiOP (ND)	[70]
Portugal	fat tissue	12	DMP (<LOD), DEP (<LOD), BBP (<LOD), DBP (<LOD-21.3 ± 1.7), DEHP (<LOD-62.6 ± 8.6), DNOP (<LOD-83.5 ± 11.2)	[71]
Japan	eggs	10	Egg white: DBP (<LOD-150), DEHP (50–40,000) Egg yolk: DBP (ND), DEHP (ND)	[72]
		155	DEHP: egg white (10–410, mean: 210 or 20–380, mean: 150 ***), yolk (ND) and shell (610–2760, mean:1730 or 900–3660, mean: 2070 ***)	
Turkey		150	Battery eggs: DMP (<LOQ-19.9, mean: 7.4), DEP (<LOQ-1.3, mean: 0.4), DBP (<LOQ-61.5, mean: 3.5), BBP(<LOQ), DEHP (<LOQ-19.8, mean: 9.5), DNOP (<LOQ-1.9, mean: 0.3) Free-range eggs: DMP (<LOQ-36.6, mean: 3.8), DEP (<LOQ-0.6, mean: 0.2), DBP (<LOQ), BBP (<LOQ), DEHP (<LOQ-13.4, mean: 3.5), DNOP (LOQ) Organic eggs: DMP (<LOQ-8.03, mean: 1.9), DEP (<LOQ-14.8, mean: 1.6), DBP (<LOQ-2.9, mean: 0.4), BBP (<LOQ), DEHP (<LOQ-19.8, mean: 7.6), DNOP (LOQ)	[18]
**PHTHALATE METABOLITES**				
**COWS**				
China	urine	100	PA (2.5–133, median: 22), MEP (ND-9.8, median: 2.7), MBP (1.8–542, median: 18), MiBP (0.44–280, median: 4.3), MBP (ND-6.9, median: 1.8), MEHP (<LOQ−43, median: 9.7), MEHHP (0.17−8.6, median: 1.8), MEOHP (<LOQ-3.8, median: 0.93), MECPP (0.34–7.7, median: 1.3), MiNP (0.1–3.8, median: 1.0); MCPP (<LOQ-7.1, median: <LOQ)	[51]
India		45	PA (ND-68, median: 8.8), MEP (1.6–212, median: 15), MBP (0.15–38, median: 1.9), MiBP (0.61–39, median: 2.8), MBP (ND-2.0, median:0.62), MEHP (2.6–92, median: 7.9), MEHHP (0.37–26, median: 1.8), MEOHP (0.07–4.9, median: 0.66), MECPP (0.26–8.1, median: 0.9), MiNP (0.15–7.6, median: 0.73), MCPP (ND-4.5, median: <LOQ)	
USA		38	PA (ND-47, median: 1.3), MEP (ND), MBP ****, MiBP (ND-0.79, median: 0.21), MBP (ND-1.4, median: 0.19), MEHP (ND), MEHHP (ND-1.9, median: 0.19), MEOHP (ND), MECPP (ND), MiNP (0.03–0.71, median: 0.3), MCPP ****	
Kyrgyzstan	hair	48	MMP (<LOD-76.5, median: <LOD), MEP (<LOD), MiBP (<LOD-469.3, median: <LOQ), MBP (<LOD-164.3, median: <LOQ,), MBP (<LOD-15.2, median:<LOD); MCHP (<LOD), MEHP (<LOD-128.3, median: 24.65)	[56]
**PIGS**				
China	liver, heart, lung, kidney, muscle stomach, pancreas spleen	3	MEHHP, MEOHP, MBP, MNOP, MEHP, MBP, MEP, MMP. Mean concentration of all mentioned above substances ranged from 48 in the heart to 108 in the liver. *****	[19]
	liver	16	MMP (<LOQ), MEP (1.2–10.8, mean: 4.2 ± 2.8), MBP (<LOQ-79.3, mean: 55.1 ± 18.4), MEHP (<LOQ-57.3, mean: 26.4 ± 14.6), MEOHP (1.6–19.2, mean: 8.2 ± 5.5)	[69]
	lung		MMP (0.4–2.0, mean: 0.9 ± 0.4), MEP (1.4–5.9, mean: 3.1 ± 1.2), MBP (22.5–40.6, mean: 31.9 ± 5.2), MEHP (10.2–33.2, mean: 22.2 ± 6.5), MEOHP (<LOQ)	
	muscle		MMP (3.2–7.7, mean: 5.3 ± 1.6), MEP (1.0–2.9, mean: 1.9 ± 0.7), MBP (26.0–70.9, mean: 47.0 ± 13.6), MEHP (17.5–34.3, mean: 24.6 ± 5.7), MEOHP (<LOQ)	
	spleen		MMP (<LOQ-2.5, mean: 0.6 ± 0.7), MEP (<LOQ-1.4, mean: 0.9 ± 0.4), MBP (11.6–16.4, mean: 13.6 ± 1.3), MEHP (9.0–34.7, mean: 11.7 ± 6.0), MEOHP (<LOQ)	
	kidney		MMP (3.5–9.4, mean: 5.6 ± 1.3), MEP (2.9–4.8, mean: 3.9 ± 0.5), MBP (16.9–332.1, mean: 24.1 ± 3.5), MEHP (7.9–19.2, mean: 12.9 ± 3.4), MEOHP (<LOQ)	
	heart		MMP (<LOQ), MEP (<LOQ), MBP (3.8–8.1, mean: 6.1 ± 1.0), MEHP (0.7–7.3, mean: 3.8 ± 1.5), MEOHP (<LOQ)	

Data are expressed as mean ± standard deviation, unless stated otherwise. * depending on grazing and milking time. ** depending on grazing time. *** depending on the frequency of egg sampling for analysis. **** Substances designated with an asterisk were present in less than 50% of the samples and were there-fore excluded from the statistical analysis by the authors. ***** The authors do not provide the exact concentrations of the individual substances tested in each organ. n–number of samples; ND—not detected; <LOD–below limit of detection; <LOQ–below limit of quantification.

**Table 2:** Summary of current knowledge on the adverse effects of PAEs and their metabolites on livestock organs and systems.

Substance	Dose or Concentration	System/Organ	Effects	Evidence Type	Ref.
**COWS**					
DEHP	100 mg/kg	female reproductive system	Hormone levels changes; Changes in ovarian follicles	In vivo	[91]
	1, 3, 10, 30 µM	female reproductive system	Alterations in luteal steroidogenesis	In vitro	[82]
	0–6, 13 × 10^−5^ mol/L	thymus	DNA alteration induction	In vitro	[90]
DEHP, MEHP	0.1, 1, 10 ng/mL	female reproductive system	Changes in secretion of prostaglandins (F2alpha and E2) and oxytocin	In vitro	[83]
MEHP	20–1000 nM	female reproductive system	Gene expression alterations; Influence on oocyte maturation; Apoptosis	In vitro	[15]
	20 nM	female reproductive system,	Gene expression alterations; Changes in cell proteins	In vitro	[16]
	50 μM	female reproductive system	Oxidative stress induction; Gene expression alterations; Mitochondrial damage; Apoptosis; mRNA expression alterations	In vitro	[79,80]
	10, 25, 50, 75 or 100 μM	female reproductive system	Influence on oocyte development	In vitro	[81]
	10^−4^ to 10^−6^ M	male reproductive system	Apoptosis	In vitro	[85]
DBP, MBP	0.19–133 μg/mL	lungs	Inhibition of superoxide dismutase activity	In vitro	[89]
DEHP, DBP, BBP	10^−9^–10^−5^ M	male reproductive system	Gene expression alterations; Apoptosis	In vitro	[84]
DEP, DPP, DBP, BBP, DnPP, DHP, DCHP, DEHP, MBP, MBzP, PA	1–1000 μM	adrenal glands	Calcium signaling suppression	In vitro	[86,87,88]
**GOATS**					
DEHP	10, 20, 40, 60, and 80 μmol/L	mammary gland	Mitochondrial damage; Oxidative stress induction; Gene expression alterations Apoptosis	In vitro	[92]
MEHP	100, 1, 1 × 10^−3^ nmol/mL	male reproductive system	Mitochondrial damage; Apoptosis	In vitro	[93]
PAE mixture	100 mg/kg	overall physiological status	Changes in physiological parameter, hormone levels, hemato-biochemical parameters	In vivo	[94,95]
**HORSES**					
DEHP	0.12, 12 1200 µM	female reproductive system	Apoptosis; Oxidative stress induction	In vitro	[96]
**PIGS**					
MBP	25–200 µM	female reproductive system	Epigenetic modifications; Changes in DNA, mRNA and protein expression; Mitochondrial damage, apoptosis	In vitro	[97,98]
BBP	10, 50, and 100 μM	female reproductive system	Mitochondrial damage; Oxidative stress induction; Disturbances in meiosis	In vitro	[99]
BBP	10, 50 and 100 μM	embryo	Development inhibition Mitochondrial damage; Oxidative stress induction, apoptosis	In vitro	[102]
BBP+ bisphenol A	10^−10^ to 10^−4^ M	female reproductive system	Inhibition of cumulus expansion, oocyte meiotic maturation, and progesterone production	In vitro	[100]
DEHP, BBP	100 pM–100 μM	female reproductive system	Impairment of cumulus expansion; gene expression alterations; Hormone alterations	In vitro	[101]
MEHP	50, 75 and 100 μM	female reproductive system	Meiotic disruption induction; Oxidative stress induction	In vitro	[104]
DiNP	25, 50 and 100 μM	female reproductive system	Meiosis inhibition; Mitochondrial damage; Oxidative stress induction, apoptosis	In vitro	[105]
DiNP, DNOP, DiDP	10^–8^–10^–4^ M	female reproductive system	Hormonal activity changes	In vitro	[106]
DBP	1.8 μM	male reproductive system	Apoptosis	In vitro	[108]
DEHP	250 μM–5 mM	female reproductive system	Disturbances in oocyte maturation	In vitro	[103]
	100–3200 μM	aortic valve	Apoptosis; Gene expression alterations	In vitro	[109]
	20, 200 mg/kg	male and female reproductive system	Changes in testis and ovaries; Hormone alterations	In vivo	[110]
	300 mg/kg	male reproductive system	Precocious maturity of bulbourethral glands; Hormone alterations; Decrease in total sperm motility; Changes in spermatozoa	In vivo	[111,112,115,116]
	50 mg/kg	male reproductive system	Hormone alterations; Changes in Leydig cells	In vivo	[117]
**SHEEP**					
DEHP	0.5 μM	female reproductive system	Oxidative stress induction	In vitro	[118]
DEHP	25, 50 mg/kg	female reproductive system	Estrus cycle disturbances; Changes in corpus luteum Hormone alterations	In vivo	[119]
DEHP	50 mg/kg	female reproductive system	Hormone alterations	In vivo	[120]
PAE mixture	Environmental exposure	Overall physiological status Hypothalamus Adipose tissue	Offspring body weight decrease; Gene expression alterations; mRNA expression alterations	In vivo	[121]
**POULTRY**					
DEHP	500 μM	heart	Protein expression alterations; Apoptosis; Necroptosis; Gene expression alterations; mitochondrial damage; mRNA expression alterations	In vitro	[122,123,124]
	60 µM	macrophages lymphocytes	Pyroptosis; Necroptosis; Oxidative stress induction	In vitro	[125]
	12.5–800 μmol/L	lymphoblasts	Oxidative stress induction	In vitro	[126]
	150 mg/kg	overall physiological status and female reproductive system	Feed intake reduction; Alterations in eggs; Ovarian follicular atresia induction; Apoptosis; Metabolism alterations	In vivo	[128]
	2% in food	overall physiological status	Lipid metabolism alterations	In vivo	[127]
	1, 3 g/kg	overall physiological status	Growth inhibition; Feed intake reduction	In vivo	[72]
	400–1600 mg/kg	spleen	Apoptosis; Inflammatory processes	In vivo	[125]
	500 mg/kg	bursa of Fabricius	Apoptosis	In vivo	[126]
	750 mg/kg	duodenum	Necrosis; Pyroptosis	In vivo	[130]
	750 mg/kg	liver	Apoptosis; Gene expression alterations; Oxidative stress induction	In vivo	[131]
DEHP, DBP	20 to 100 mg/kg	embryo	Embryotoxic effect	In ovo	[134]
Phthalate monoesters mixture	85 µg/g egg	embryo	Embryotoxic effects; Oxidative stress induction; Apoptosis	In ovo	[136]

## Data Availability

No new data were created or analyzed in this review study. Data sharing is not applicable to this article.

## Supplementary Materials

The following supporting information can be downloaded at: https://www.mdpi.com/article/10.3390/ijms27198746/s1.
