# Supplementary material for: Phthalates in Animal Husbandry—A Narrative Review
Source: Int J Mol Sci. 2026 Sep 30;27(19):8746. doi: 10.3390/ijms27198746 (PMC13665416; doi:10.3390/ijms27198746)

Supplementary materials to

Phthalates in animal husbandry – a narrative review

Slawomir Gonkowski

Department of Clinical Physiology, Faculty of Veterinary Medicine, University of Warmia and Mazury in Olsztyn, Oczapowskiego 13, 10-957 Olsztyn, Poland.

Electronic address: slawomir.gonkowski@uwm.edu.pl.

Table S1 Commonly Used Industrial Phthalates and Their Primary Metabolites

Phthalates (Acronym)	Molecular Formula	R1	R2	Metabolites (Acronym)
SHORT ALKYL CHAIN PHTHALATES				
Dimethyl phthalate (DMP)	$C_{10}H_{10}O_4$	CH_3	CH_3	Monomethyl phthalate (MMP)
Diethyl phthalate (DEP)	$C_{12}H_{14}O_4$	CH_2CH_3	CH_2CH_3	Monoethyl phthalate (MEP)
Diisobutyl phthalate (DiBP)	$C_{16}H_{22}O_4$	$CH_2CH(CH_3)_2$	$CH_2CH(CH_3)_2$	Monoisobutyl phthalate (MiBP)
Dibutyl phthalate (DBP)	$C_{16}H_{22}O_4$	$CH_2CH_2CH_2CH_3$	$CH_2CH_2CH_2CH_3$	Mono(n-butyl) phthalate (MBP)
Bis(2-methoxyethyl) phthalate (DMEP)	$C_{14}H_{18}O_6$	$CH_2CH_2OCH_3$	$CH_2CH_2OCH_3$	Mono(2-methoxyethyl) phthalate (MMEP)
Dicyclohexyl phthalate (DCHP)	$C_{20}H_{26}O_4$	C_6H_{11}	C_6H_{11}	Monocyclohexyl phthalate (MCHP)
Di(n-pentyl) phthalate (DnPeP)	$C_{18}H_{26}O_4$	$CH_2(CH_2)_3CH_3$	$CH_2(CH_2)_3CH_3$	Mono(n-pentyl) phthalate (MnPeP)
Diallyl phthalate (DAP)	$C_{14}H_{14}O_4$	C_3H_5	C_3H_5	Monoallyl phthalate (MAP)
Di(n-propyl) phthalate (DPP)	$C_{14}H_{18}O_4$	C_3H_7	C_3H_7	Mono(n-propyl) phthalate (MnPP)
LONG ALKYL CHAIN PHTHALATES				
Benzyl butyl phthalate (BBP)	$C_{19}H_{20}O_4$	$CH_2C_6H_5$	C_4H_9	Monobenzyl phthalate (MBzP)
Diphenyl phthalate (DPhP)	$C_{20}H_{14}O_4$	C_6H_5	C_6H_5	Monophenyl phthalate (MPhP)
Di(2-ethylhexyl) phthalate (DEHP)	$C_{24}H_{38}O_4$	$CH(CH_2)_5(CH_3)_2$	$CH(CH_2)_5(CH_3)_2$	Mono(2-ethylhexyl) phthalate (MEHP)<br>Mono(2-ethyl-5-hydroxyhexyl) phthalate (MEHHP)<br>Mono(2-ethyl-5-oxohexyl) phthalate (MEOHP)<br>Mono(2-ethyl-5-carboxypentyl) phthalate (MECPP)
Di(n-octyl) phthalate (DNOP)	$C_{24}H_{38}O_4$	$(CH_2)_7CH_3$	$(CH_2)_7CH_3$	Mono(n-octyl) phthalate (MNOP)<br>Mono(3-carboxypropyl) phthalate (MCPP)
Diisononyl phthalate (DiNP)	$C_{26}H_{42}O_4$	$CH(CH_2)_6(CH_3)_2$	$CH(CH_2)_6(CH_3)_2$	Monoisononyl phthalate (MiNP)
Di(n-nonyl) phthalate (DnNP)	$C_{26}H_{42}O_4$	$(CH_2)_8CH_3$	$(CH_2)_8CH_3$	Mono(n-nonyl) phthalate (MnNP)

Figure S1 Phthalate metabolism

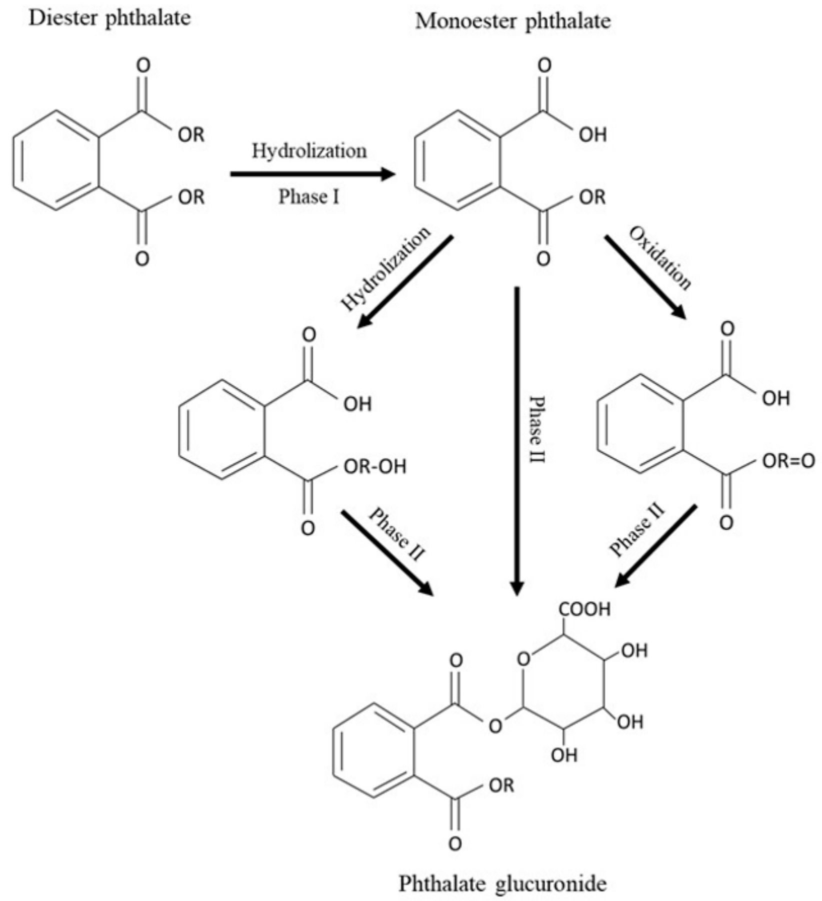

Supplement: Supplementary file 1 [file ijms-27-08746-s001.zip › ijms-4537114-supplementary.pdf]
